# Intrinsic Disorder as a New Frontier in Antimicrobial Resistance and Bacterial Fitness

**DOI:** 10.1002/mbo3.70415

**Published:** 2026-09-08

**Authors:** Jack O' Callaghan, Michael P. Ryan, Sarah Hudson, Damien Thompson

**Affiliations:** ^1^ Department of Physics University of Limerick Limerick Ireland; ^2^ Bernal Institute University of Limerick Limerick Ireland; ^3^ Research Ireland Centre for Pharmaceuticals (SSPC) University of Limerick Limerick Ireland; ^4^ Department of Chemical Sciences University of Limerick Limerick Ireland

**Keywords:** antimicrobial resistance, bacterial fitness, IDPs, intrinsic disorder

## Abstract

Intrinsically disordered proteins (IDPs) are biological macromolecules lacking a well‐defined structure under normal physiological conditions. These proteins can be essential for both eukaryotic and prokaryotic cells, yet bacterial IDPs are significantly understudied. In this review, we highlight the functional importance of intrinsic disorder across a range of microbial phenomena, including effector secretion, desiccation tolerance, and the bacterial stress response. Given the emergence of structural disorder in key mechanisms of bacterial fitness, we propose bacterial intrinsic disorder as a novel linkage to antimicrobial resistance (AMR). We highlight areas of research where further study of bacterial IDPs could provide new insights into microbial evolution. Functional disorder introduces a new paradigm to the challenge of AMR, identifying novel targets for antimicrobial therapy.

## Graphical abstract

The works examines the link between intrinsic disorder in bacterial protein structure, and bacterial function, and antimicrobial resistance. It highlights how flexible, disordered proteins support secretion, stress responses, desiccation protection, and virulence, while also suggesting that these adaptable regions may provide novel targets for antimicrobial therapy.

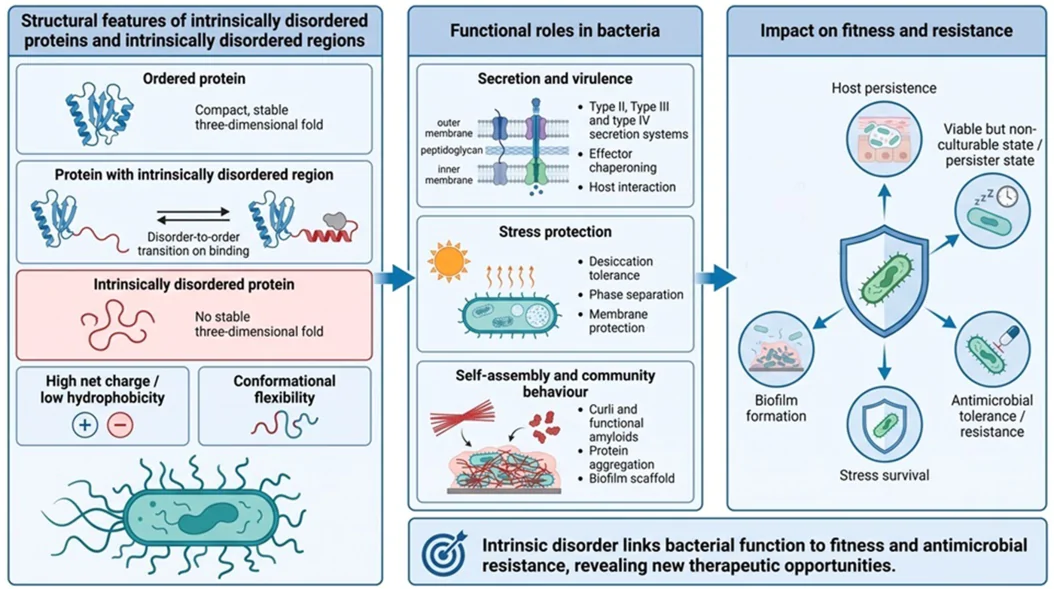

## 1 Introduction

**Figure 1:**
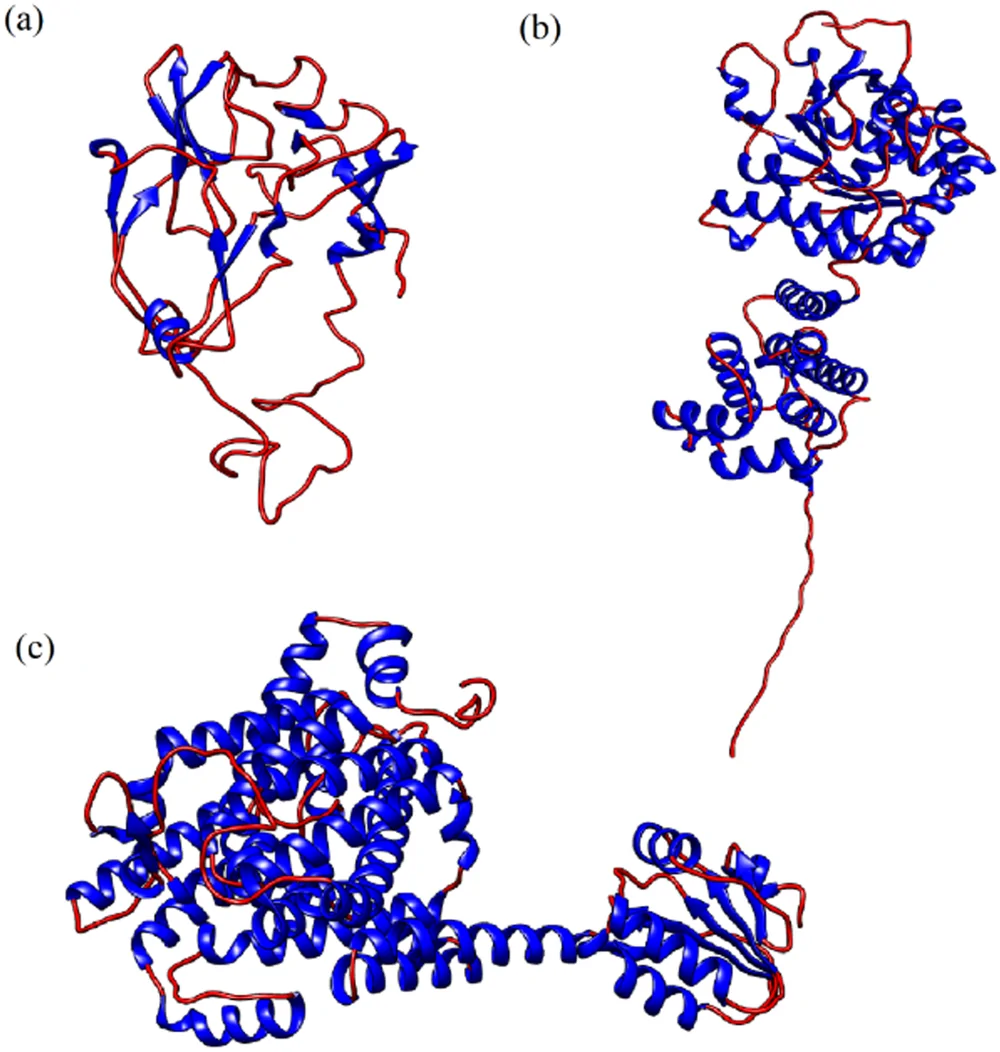
An example of (a) a bacterial intrinsically disordered protein (IDP), the *B. subtilis* 50S ribosomal protein, *rplB* (IDP, PDB accession: 7AQC—rplB bound to the bacterial ribosome), (b) a bacterial protein with an intrinsically disordered region (IDR), the *Escherichia coli* K12 replicative DNA helicase, *dnaB* (C‐terminus IDR, AlphaFold predicted (Jumper et al. 2021), and (c) an ordered bacterial protein, *E. coli* K12 C4‐dicarboxylic acid transporter, *dauA* (AlphaFold predicted (Jumper et al. 2021). Secondary structures are coloured blue, unstructured regions are red.

Intrinsically disordered proteins (IDPs) challenge the classical structure–function paradigm of biochemistry that a string of amino acids, encoded by DNA, folds to yield a unique, energetically stable protein equilibrium structure. This “structure–function” archetype implies that a folded macromolecular structure with a minimum free energy is required for biological function (Wright and Dyson 1999). By contrast, the demonstration of biological activity in the absence of a well‐defined 3D assembly is a distinguishing feature of functional IDPs, whose inherently unstructured conformations confer an innate versatility and adaptability that influences cell signalling, cell regulation and substrate recognition (Uversky 2014; Cohan and Pappu 2020). Proteins can be classified as IDPs if they do not adopt a well‐defined secondary or tertiary structure under normal physiological conditions (Figure 1) (van der Lee et al. 2014).

An example of a disordered bacterial protein is FapA, a periplasmic chaperone for the functional amyloid FapC in *Pseudomonas aeruginosa*. The disordered N‐terminus of FapA facilitates complexation with pre‐amyloidal FapC, protecting the cell against FapC fibrillation (Rasmussen et al. 2023). Similar to FapA, many globular proteins contain polypeptide segments that do not form a well‐defined structure yet are still functional. These intrinsically disordered regions (IDRs) include many charged amino acid residues, giving a low mean hydrophobicity. In the absence of specific macromolecular interaction partners that can assist with folding in vivo (typically other proteins, RNA molecules or biological interfaces) intramolecular electrostatic repulsion prevents folding under normal physiological conditions (Uversky 2011). As a result, the free energy landscapes of disordered proteins are relatively flat, unlike ordered proteins that have a single well‐resolved minimum energy structure (Chong et al. 2019).

**Figure 2:**
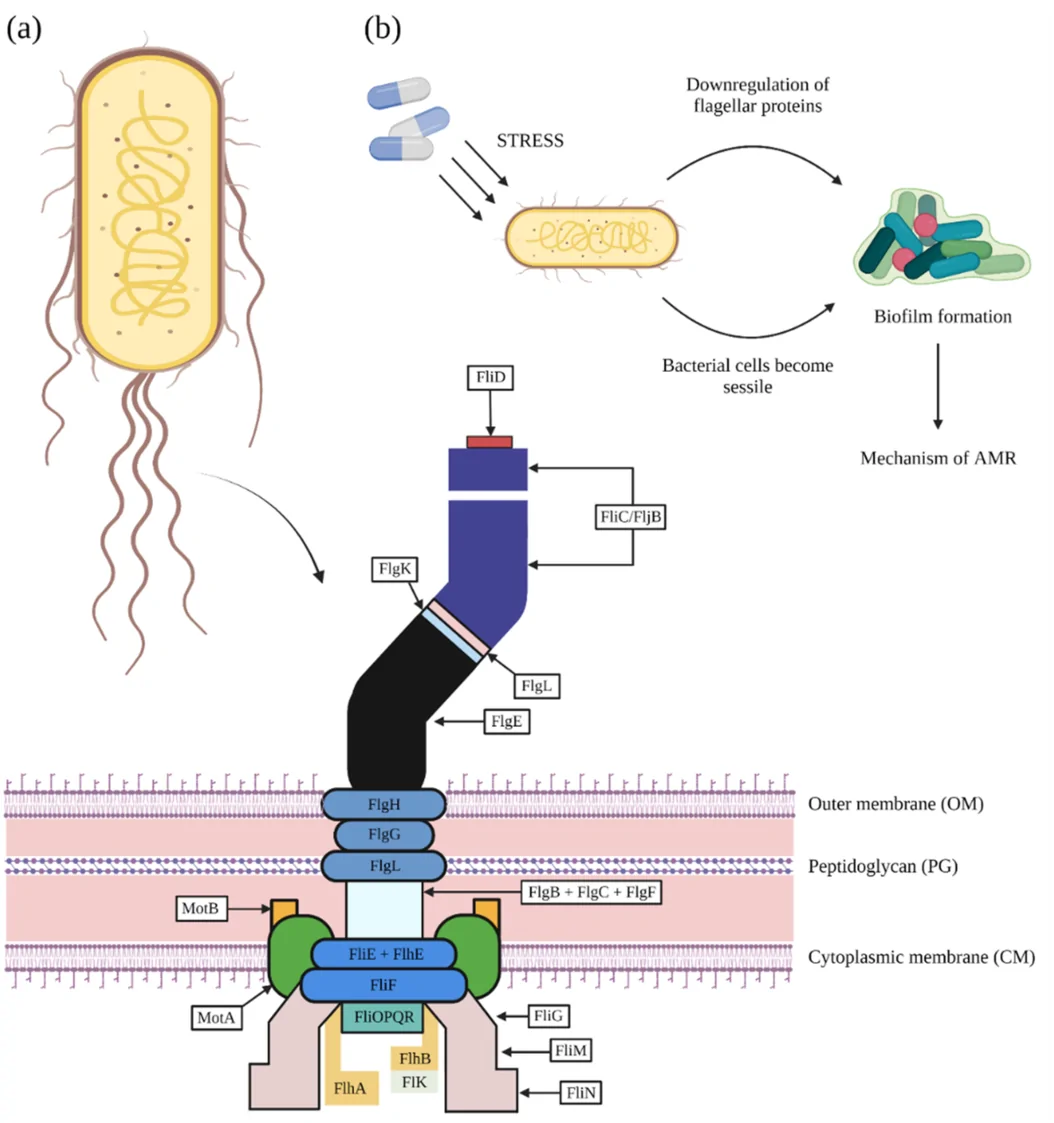
(a) Intrinsically disordered proteins (IDPs) comprise both the structural components of the bacterial flagella (FliK (Kinoshita et al. 2017) and FliM (Minamino et al. 2019)) and are involved in the regulation of its assembly (FliC (Molinari et al. 2025) and FlgM (Dedmon et al. 2002)) (b) Antibiotic‐induced stress induces the downregulation of flagellar and motility proteins linked to microbial sessility, preceding biofilm formation.

IDPs have been extensively studied in eukaryotes, particularly as targets for neurodegeneration and cancer (Uversky et al. 2008). Over one third of eukaryotic proteins are predicted to contain “long” IDRs (defined by a continuous IDR greater than thirty amino acids in length) compared to less than 5% in bacterial proteomes (Ward et al. 2004). This cut‐off for the definition of a “long” IDR is well‐established in the literature and serves to identify meaningfully long IDRs (Xue et al. 2009). It has also been used to account for protein regions that may not appear in X‐ray crystallographic data (by virtue of being disordered) that may yet be functionally important (Monzon et al. 2020). The low incidence rate of prokaryotic IDPs observed using the above classification system is mirrored in a range of other schemes, including classifying IDPs based on the fraction of disordered residues in the protein's primary structure, or by the presence of tandem repeats that are overrepresented in IDRs (van der Lee et al. 2014). In a previous work, we applied the length‐based classification scheme above to 58 bacterial proteomes from clinically relevant strains, confirming an IDP abundance of 3%–7% (O'Callaghan et al. 2024). Despite this lower incidence, protein disorder has been shown to be important for a broad range of functions in bacteria, including plasmid‐mediated phenomena (Mitić et al. 2018), virulence factor secretion (Van Gerven et al. 2018), the expression of effector proteins (Marín et al. 2013), and key structural functions (Shanmugam et al. 2019) (see Figure 2). In this review, the importance of functional disorder in bacteria is presented, its biological significance is highlighted, and the implications of this disorder for various phenomena (including microbial pathogenesis and the bacterial stress response) are discussed.

## 2 Intrinsically Disordered Proteins Enable Bacterial Pathogenicity and Virulence

Rather than acting as passive structural elements, intrinsically disordered proteins (IDPs) and intrinsically disordered regions (IDRs) contribute to bacterial virulence through a limited set of recurring functional mechanisms. We summarise below well‐defined examples of bacterial IDPs (or related phenomena) fulfilling specific functions related to bacterial secretion systems. For the T2SS, it is the use of disorder‐to‐order transitions in secretin transport. For the T3SS, it is the translocated intimin receptor (Tir) and how it interacts with the host immune system to facilitate infection. For the T4SS we discuss plasmid‐encoded proteins, and the disordered interactions involved in toxin‐antitoxin binding.

### 2.1 Disorder‐to‐Order Transitions in Secretion and Binding

A defining feature of IDPs is their ability to undergo coupled folding and binding, enabling tightly regulated molecular interactions (Chong et al. 2019). In the Type‐II secretion system (T2SS), intrinsic disorder contributes to secretin assembly and gating. Disordered regions enable conformational sampling and undergo disorder‐to‐order transitions during secretin oligomerisation, facilitating the regulated export of folded proteins (Korotkov et al. 2012; Gu et al. 2017). IDRs also support membrane association and substrate positioning for secretion (Gu et al. 2017). While disorder is a well‐established component of secretory phenomena, it is understudied in the T2SS. A comparable mechanism occurs in Type‐IV secretion system (T4SS)‐associated plasmid proteins, particularly toxin–antitoxin systems. Intrinsically disordered C‐terminal domains of antitoxins fold upon binding their cognate toxins, ensuring specificity and tight regulatory control (Mitić et al. 2018). This illustrates how disorder‐driven transitions underpin plasmid‐mediated processes linked to antimicrobial resistance (Bennett 2008; Ryan et al. 2024).

### 2.2 IDPs as Chaperones and Stabilisation Factors

Intrinsic disorder frequently enables chaperone‐like behaviour, particularly in secretion pathways requiring substrate stabilisation prior to export. In the T3SS, many effectors contain disordered regions that mediate interactions with secretion chaperones (Chen et al. 2006; Marín et al. 2013). The translocated intimin receptor (Tir) provides a clear example: its intrinsically disordered N‐terminal region binds a chaperone that promotes stabilisation, accumulation, and efficient translocation through the secretion apparatus (Chen et al. 2006; Vieira et al. 2024). Similarly, in the T2SS, intrinsic disorder contributes to the correct trafficking and sorting of pilotin lipoproteins involved in secretin assembly. Disorder is a characteristic feature of the Lol lipoprotein sorting system which enhances interaction versatility, supporting efficient targeting to the outer membrane (El Rayes et al. 2021). These observations align with broader evidence that IDPs can function as molecular chaperones, stabilising aggregation‐prone or secretion‐competent substrates (Chakrabortee et al. 2011; Rasmussen et al. 2023).

### 2.3 Conformational Flexibility in Secretion and Host–Pathogen Interactions

A key advantage of intrinsic disorder is conformational flexibility, which facilitates both secretion and interaction with host targets (Marín et al. 2013; Cohan and Pappu 2020). T3SS effectors are often enriched in disordered regions, which may allow them to remain partially unfolded during translocation through the narrow secretion channel (Hamada et al. 2010; Yan and Bassler 2019). Once inside the host cell, this flexibility enables binding to multiple partners. For example, Tir interacts with intimin and host cytoskeletal components to promote actin pedestal formation, enhancing adhesion and resistance to phagocytosis (Ruchaud‐Sparagano et al. 2011; Battle et al. 2014; Velle and Campellone 2017). Beyond structural adaptability, IDRs enable immune modulation. Tir suppresses immune signalling pathways, including NF‐κB activation, illustrating how flexible protein regions facilitate interactions with diverse host regulatory proteins (Ruchaud‐Sparagano et al. 2011; Vieira et al. 2024). In plasmid‐associated systems linked to the T4SS, intrinsic disorder similarly supports interaction promiscuity. Though not extensively characterised, many plasmid‐encoded proteins are enriched in disorder (Mitić et al. 2018), enabling participation in diverse processes including conjugation, antimicrobial resistance, and biofilm formation (Bennett 2008; Yan and Bassler 2019; Ciofu et al. 2022).

### 2.4 Implications for Pathogenicity and Therapeutic Targeting

Across secretion systems, intrinsic disorder provides a unifying functional advantage: structural adaptability combined with interaction versatility (Wright and Dyson 1999; Uversky 2011; Cohan and Pappu 2020). These properties enable efficient secretion, adaptive binding, and regulatory control, all of which are central to bacterial virulence. This mechanistic perspective has implications for antimicrobial development. Secretion systems are established drug targets (Waack et al. 2017), and targeting intrinsically disordered regions could disrupt multiple pathways simultaneously, including effector stabilisation, secretion, and key host interactions.

## 3 Disordered Proteins Regulate Key Mechanisms of Bacterial Fitness

Environmental stressors, deriving from host immune pressures or the action of antimicrobial compounds, present a constant threat to pathogens in healthcare settings. The success of nosocomial contagions in a clinical environment, particularly ESKAPEE pathogens (Miller and Arias 2024) (*Escherichia coli*, *Staphylococcus aureus*, *Klebsiella pneumoniae, Acinetobacter baumannii, Pseudomonas aeruginosa, Enterococcus faecium* and Enterobacter species) rests on two key factors: the activation of multi‐drug resistance pathways, and tolerance to desiccation and ethanol (Farrow et al. 2018). Desiccation is characterised by an acute loss of water in the microorganism, which can denature proteins, generate DNA‐damaging reactive oxygen species, and break the cell wall (Chakrabortee et al. 2011). As summarised in Table 1, several bacterial desiccation tolerance mechanisms are heavily reliant on the action or aggregation of disordered proteins.

**Table 1:** Functional disorder in evolved mechanisms of bacterial desiccation tolerance.

Mechanism	Brief description	IDP(s)	Functional significance	Refs
Phenotypic differentiation	Extremophilic bacteria, e.g., xerophiles differentiate into spores or cysts to facilitate microbial dormancy	Hydrophilins	Short IDPs termed **l** ate **e** mbryogenesis **a** bundant (LEA) proteins regulate cyst desiccation	(Lebre et al. (2017); Rodriguez‐Salazar et al. (2017))
Biofilm and spore formation	Biofilm formation sequesters water into the local microenvironment improving survival rates under dry conditions	Outer membrane proteins, functional amyloids	Disordered proteins maintain biofilm integrity, stabilising the extracellular matrix and improving tolerances to environmental and abiotic stresses	(Taglialegna et al. (2016); Van Gerven et al. (2018); Yan and Bassler (2019))
Upregulation of stress and DNA‐repair proteins	Circumvents reactive oxygen‐induced DNA damage in bacteria	Hydrophilins	LEA proteins act as stress effectors, mitigating against the effects of extreme drying	(Koshland and Tapia (2019))
Cytosolic glass‐transition	Diminished metabolic activity in bacterial dormancy leads to decreased cytoplasmic fluidity, stabilising the cell wall	Unknown	Correlation between expression of IDPs and evolution of glass‐transition phenomena in *eukaryotes*, specifically tardigrades^a^	(Parry et al. (2014); Boothby et al. (2017); Laskowska and Kuczyńska‐Wiśnik (2020))

^a^ Tardigrades are microscopic invertebrates with an IDP‐reliant cytoplasmic vitrification mechanism.

**Figure 3:**
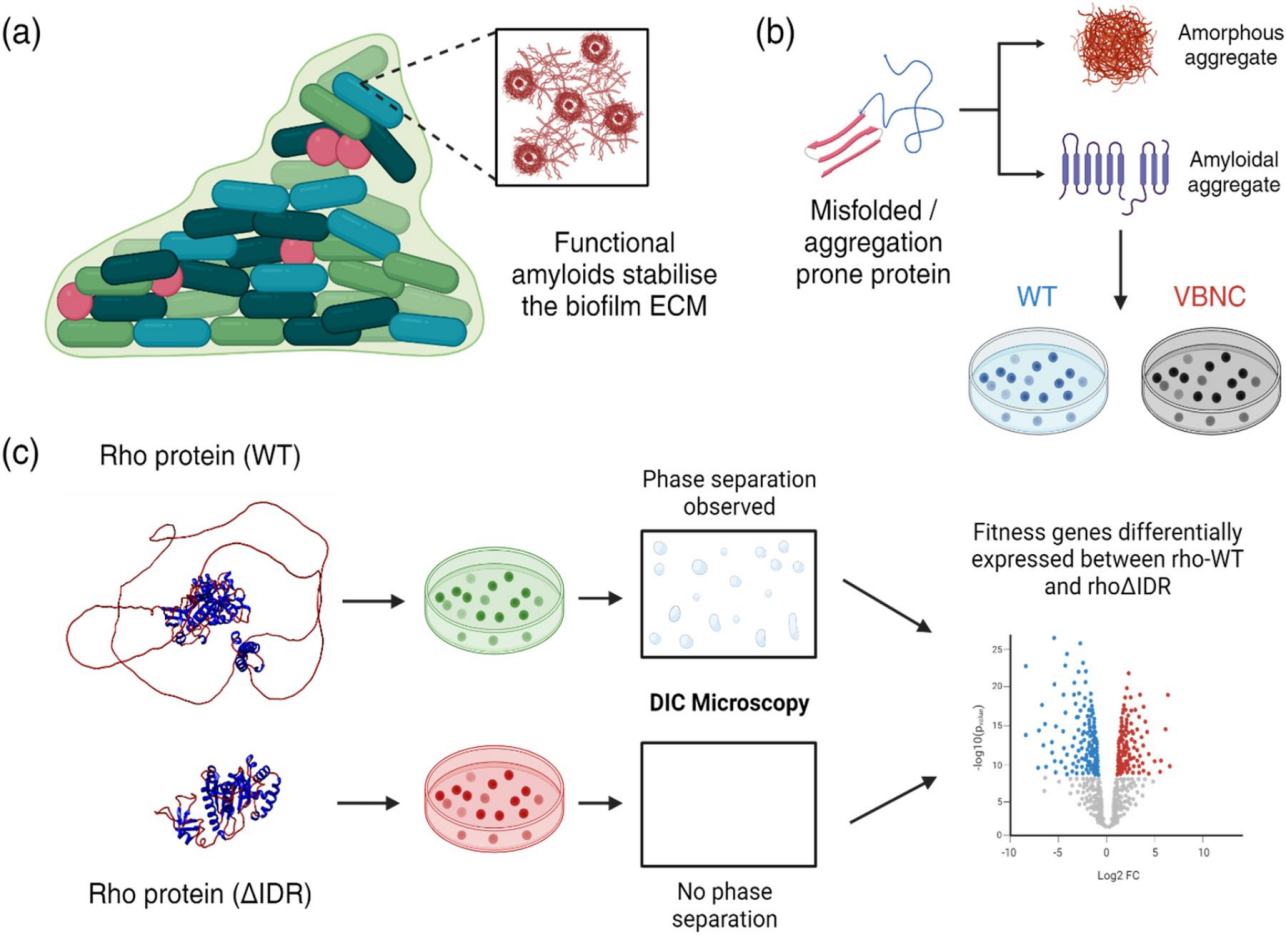
(a) Functional amyloids confer stability to the extracellular matrix (ECM) of bacterial biofilms (Yan and Bassler 2019), (b) Misfolded or aggregation prone regions (APRs) in bacterial proteins can evolve the amorphous or amyloidal aggregates that accompany viable but non‐culturable (VBNC) cell populations (Bollen et al. 2021), (c) The Rho protein of *Bacteroides thetaiotaomicron* is essential for phase separation, and promotes fitness genes in an IDR‐reliant manner (Krypotou et al. 2023) (WT—wild type, ΔIDR intrinsically disordered region knockout, DIC—differential interference contrast).

Mitigating against the effects of abiotic stress and nutrient deprivation is a crucial feature of bacterial fitness, which is commonly achieved *via* desiccation tolerance. Protein phase separation, a key driver in the formation of membrane‐less organelles, underlies biological functions important for stress biology. Many of the proteins that mediate this phase separation have functionally significant IDRs (Ibrahim et al. 2023). A recent study of the Gram‐negative bacteria *Bacteroides thetaiotaomicron* uncovered an IDR‐centric phase separation mechanism that promotes bacterial fitness in the mammalian gut (Krypotou et al. 2023). This mechanism relies on the transcription termination factor Rho which is essential for bacterial cell viability (Banerjee et al. 2006). The *B. thetaiotaomicron* Rho protein has a long IDR that regulates its phase separation into a membrane‐less compartment (Krypotou et al. 2023). Though not applicable to the Rho‐factors of all bacterial species, the disorder‐based mechanism utilised by these bacteria to survive a harsh host environment illustrates the broader functions of IDRs in a pathogenic context (see Figure 3).

Biofilms reflect a common phenotype of desiccation‐tolerant bacteria across a diverse range of ecosystems (Ciofu et al. 2022). They serve a protective role in extreme, nutrient‐depleted environments by helping to retain water under dry conditions and providing a polymeric “shield” against external stressors such as antibiotics. The structural and adhesive properties of the biofilm can be influenced by the expression of IDPs, including functional amyloids like the major curlin subunit (*csgA*) in *E. coli*. Curli fibrils are extracellular fibrils that resemble the human pathological amyloids in neurodegenerative diseases such as Alzheimer's. Bacterial amyloids are extensively disordered in their native monomeric state, even though the final, assembled form of curli fibres are highly ordered with a well‐defined β‐sheet structure. The upregulation of these IDPs manifests as desiccation tolerance, and amyloidal architectures provide bacterial colonies with an ideal scaffold for biofilm formation (Perov et al. 2019). Desiccation tolerance is a characteristic feature of curliated bacteria, and microbes with an upregulated expression of amyloid‐encoding genes are innately resistant to drying (Shanmugam et al. 2019).

The prevalence of these functional amyloids in the structure and biogenesis of the microbial biofilm supports a possible link between intrinsic disorder and antimicrobial resistance. For example, downregulation of flagellar and motility proteins in carbapenem‐resistant *K. pneumoniae* has been shown to arbitrate the immobilisation of microbial cells, initiating biofilm formation (Sharma et al. 2019). Many bacterial proteins associated with flagellar assembly and motility are, by necessity to allow movement, intrinsically disordered (Barker et al. 2017; Kinoshita et al. 2017; Oguri et al. 2019) (see Figure 2). A previous study has also demonstrated that inhibition of curli proteins in *E. coli* and *B. subtilis* by the human transport protein transthyretin can prevent biofilm formation, a key resistance mechanism in Gram‐negative bacteria (Jain et al. 2017).

Late embryonic abundant (LEA) proteins are a family of biological macromolecules present in multiple organisms (predominately plants) that are known to protect cells against environmental stress. Many of these LEA proteins belong to a larger group, termed hydrophilins, that are characterised by an extensive degree of hydrophilicity and a Gly content greater than 6% (Battaglia et al. 2008). Many functionally significant LEA proteins can be described as IDPs, and the conformational freedom imposed by this disorder underpins a range of desiccation tolerance mechanisms (Artur et al. 2019). *Azotobacter vinelandii*, a Gram‐negative soil bacterium, can differentiate to form cysts. A previous study (Rodriguez‐Salazar et al. 2017) identified that cyst‐presenting *A. vinelandii* cells encode two putative tolerance genes: *lea1* and *lea2*. These were found to be essential for counteracting the effects of extreme drying and hyper‐osmolality (the effect of extracellular agents drawing water from cells by osmosis). The upregulation of these genes in *E. coli* conferred a similar protective effect. In a separate study (Gao and Lan 2016) a group of LEA genes introduced into *E. coli* enhanced resistance to both heat and osmotic stress.

## 4 The Self‐Assembly of Disordered Proteins Can Protect Bacterial Cells

Antimicrobial peptides (AMPs) form a key component of the innate immune response for multiple organisms, exhibiting potent activity against invading bacterial pathogens. In a microbiology context, research on the self‐assembly of peptides into amyloid‐like structures has been dominated by the directed synthesis of antibacterial nanomaterials (Schnaider et al. 2020). However, these same amyloidal architectures may also serve an auxiliary function as molecular shields in microbes (Chakrabortee et al. 2011). Despite cytotoxic effects linked to protein aggregation (Bhattacharya et al. 2018), IDP self‐assembly can foster the transition of bacteria from a planktonic state to microbial dormancy. The over‐expression of misfolded or aggregation‐prone proteins has been correlated with the emergence of **v** iable **b** ut **n** on **c** ulturable (VBNC) cell populations (Bollen et al. 2021). VBNC cells are less susceptible to the action of bactericidal agents, supporting several AMR mechanisms. There is also a pre‐existing relationship between VBNC dormancy and the antibiotic persister cells frequently associated with AMR evolution (Ayrapetyan et al. 2018) (see Figure 3). Not all misfolded or aggregation‐prone proteins are intrinsically disordered (Bhattacharya et al. 2018), but aggregation‐prone IDPs associated with VBNC evolution have previously been identified in *A. baumannii*—specifically the desiccation tolerance protein A, DtpA (König et al. 2023). This stress adaptation hydrophilin was found to be differentially expressed in VBNC cells and was shown to form protective aggregates under extreme environmental conditions, supporting an additional link between IDPs and AMR (Green et al. 2022).

Following antibiotic administration, the bacterial cell wall is typically subjected to external stress. Reversible self‐assembly of an inner membrane associated protein, namely, IM30 of the PspA family which was previously identified as intrinsically disordered, acts to stabilise damaged or susceptible cellular membranes in cyanobacteria (Junglas et al. 2020). This disorder is preserved at both the C‐ and N‐terminus following dimerisation, creating a film across the membrane that shields it from further damage. This mirrors the stability provided by functional amyloids in response to desiccation stress in biofilm‐forming bacteria. A similar, aggregation‐based protective effect has been reported in pathogenic yeast (Chen et al. 2021) where aggregation of various proteins provides stress resistance under damaging physiological conditions, suggesting an epigenetic explanation for the cross‐generational propagation of these aggregation‐prone proteins. Beyond self‐assembly, a balance between order and disorder in protein folding underpins mechanisms of protein‐based sensors for the identification and sequestering of antibiotics. An example of this is given by a short isoform of the Thiostrepton‐induced protein A (TipAS) in *Streptomyces lividans*. The disordered N‐terminus of this protein gains structure in the presence of thiopeptide antibiotics to facilitate antibiotic sequestration, supporting a mechanism of multi‐drug resistance (Natarajan et al. 2024).

## 5 Conclusion

Disordered proteins can serve a defensive purpose in microbial cells, protecting against various sources of cellular stress. They can also be used to counteract innate host defence mechanisms through a combination of effector translocation and signal cascade subversion. This suggests that intrinsic disorder is an important and emerging feature of bacterial adaptation, contributing to strain pathogenicity by mitigating against environmental stressors. The evidence presented here indicates that further research on the emergence of structural disorder in plasmid‐mediated phenomena, bacterial fitness, and AMR evolution could afford new insights into drug‐resistant pathogens. This would have significant implications for antimicrobial drug discovery, supporting new approaches to combat drug‐resistant bacterial infections. Specifically, the targeting of bacterial IDPs for antimicrobial therapy could yield a class of antibiotics with a mechanism of action distinct from other existing drug classes. Structured proteins are also overrepresented in traditional AMR databases—their reliance on sequence homology for the identification of AMR factors means that IDPs (or proteins enriched in IDRs) have likely been overlooked (van der Lee et al. 2014). The functional annotation of bacterial IDPs, and the incorporation of bacterial IDPs into existing AMR databases would enable a re‐analysis of existing literature through the lens of disorder, providing new insights into how protein structure interacts with host and environmental factors to support mechanisms of resistance and bacterial fitness.

## Author Contributions

**Jack O'Callaghan:** conceptualization, investigation, writing – original draft, writing – review and editing, data curation. **Michael P. Ryan:** investigation, writing – review and editing, supervision, project administration. **Sarah Hudson:** investigation, writing – review and editing, supervision, project administration. **Damien Thompson:** conceptualization, writing – review and editing, supervision, project administration.

## Ethics Statement

The authors have nothing to report.

## Conflicts of Interest

The authors declare no conflicts of interest.

## Data Availability

The authors have nothing to report.
